# Ferroptosis in hematological malignancies: molecular mechanisms and therapeutic potential

**DOI:** 10.1007/s13402-025-01142-w

**Published:** 2025-12-29

**Authors:** Jiaxi Liu, Rui Liu, Jiyu Miao, Aili He

**Affiliations:** 1 Department of Hematology, The Second Affiliated Hospital of Xi’an Jiaotong University, 157, 5th West Road, Xi’an, Shaanxi 710004 China; 2 National-Local Joint Engineering Research Center of Biodiagnostics & Biotherapy, The Second Affiliated Hospital of Xi’an Jiaotong University, Xi’an, Shaanxi 710004 China

**Keywords:** Ferroptosis, Cell death, Leukemia, Lymphoma, Myeloma

## Abstract

Ferroptosis, an iron-dependent form of regulated cell death characterized by overwhelming accumulation of lipid peroxidation, has emerged as a prominent area of interest in cancer research. Its underlying mechanisms are complex, and the high heterogeneity of hematologic malignancies adds additional challenges. Unlike solid cancers, hematologic malignancies lack fixed tissue architecture and exist within the dynamic bone marrow microenvironment, where iron metabolism, redox balance, and lipid remodeling are uniquely regulated. These differences create distinct metabolic vulnerabilities—particularly in iron and polyunsaturated fatty acid metabolism—that may render hematologic cancer cells more sensitive to ferroptotic stress. Given these unique features, a systematic understanding of ferroptosis in hematologic malignancies is critical for both elucidating disease mechanisms and exploring novel therapeutic strategies. This review summarizes the current understanding of ferroptosis in the pathogenesis and therapeutic resistance of hematologic malignancies, highlighting its mechanistic diversity across leukemia, lymphoma, and multiple myeloma. We also discuss emerging therapeutic strategies that exploit ferroptosis and outline key challenges and future directions for translating ferroptosis-based interventions into clinical practice.

## Introduction

Cell death is a fundamental biological process essential for maintaining physiological functions and cellular homeostasis [1]. Over the past decade, extensive research on various forms of regulated cell death (RCD), including apoptosis, necroptosis, pyroptosis and ferroptosis, has yielded remarkable breakthroughs. RCD eliminates aging or dysfunctional cells, preserving tissue balance, whereas dysregulation can drive uncontrolled proliferation and malignancy [2]. Ferroptosis is an iron-independent, non-apoptotic cell death identified by Dixon in 2012 [3]. It is distinguished from other types of RCD by its unique morphological characteristics and molecular mechanisms [4]. Unlike necrosis or apoptosis, which primarily target cell membrane and result in typical apoptotic body formation, ferroptosis is characterized by mitochondrial alterations, including mitochondrial membrane shrinkage, swelling, and loss of mitochondrial cristae [5].

**Figure 1:**
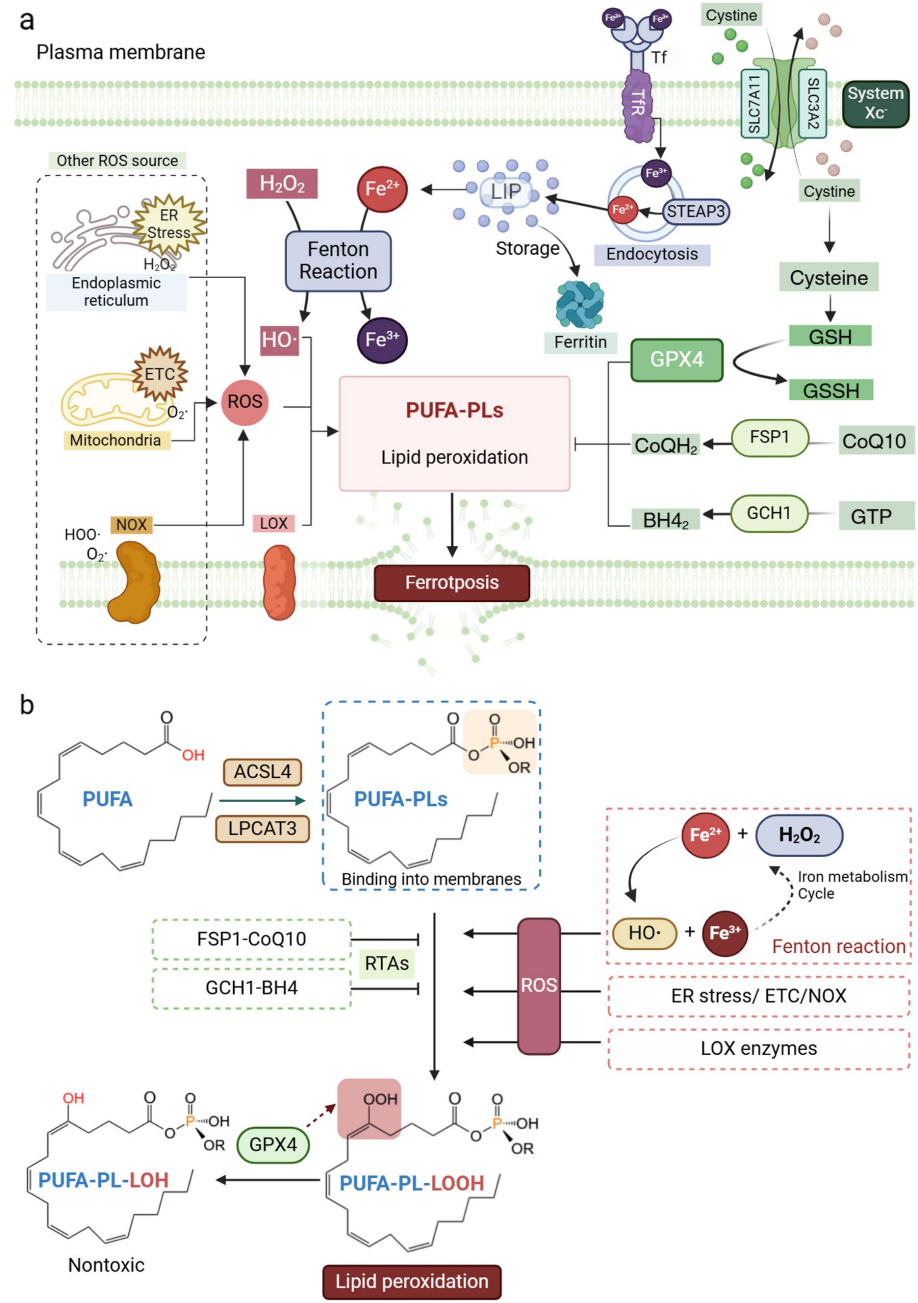
Molecular mechanisms of ferroptosis. (**a**): The biological essence of ferroptosis lies in the imbalance between lipid peroxidation and antioxidation. O_2_, PUFA-PLs and labile iron are three prerequisites for the induction of ferroptosis. O_2_ act as a form of ROS in this process. HO·, produced through Fenton reaction between H_2_O_2_ and Fe^2+^, is the most prevalent ROS initiating lipid peroxidation. Other ROS forms, such as O_2_·and HOO, generated from various sources, also contribute to lipid peroxidation. LOX enzymes, a dioxygenase containing non-heme iron, can also directly catalyze lipid peroxidation enzymatically. Iron homeostasis is maintained through transferrin-mediated iron transport, ferritin storage, and the LIP. Fe^3+^ was bind by TF and transmitted enter into cells through endocytosis after forming a complex with TfR, Fe^3+^ and then are reduced to Fe^2+^ by STEAP3. Iron is stored in the LIP in the form of Fe^2+^, and excess ion iron are sequestered by ferritin for stable storage. The system Xc^-^ composed of SLC7A11 and SLC3A2, facilitates cystine uptake for GSH synthesis, which serves as a substrate for GPX4. The GSH-GPX4 is the primary defense against ferroptosis, with additional contributions from the FSP1-CoQ10 and GCH1–BH4 pathways. (**b**): The mechanism of lipid peroxidation generation. ACSL4 and LPCAT3 catalyze the attachment of PLs to PUFAs, resulting in the formation of PUFA-PLs. The susceptibility of lipids to peroxidation depends on the strength of its carbon-hydrogen bonds. PUFAs, characterized by weak C–H bonds between adjacent C = C double bonds, are highly susceptible to peroxidation. Once integrated into cellular membranes, PUFA-PLs become primary targets for ferroptosis. ROS from various sources and LOX enzymes promote lipid peroxidation, while RTAs and GPX4 contribute to inhibiting lipid peroxidation. Notably, GPX4 plays a crucial role in reducing lipid peroxides to lipid alcohols, mitigating ferroptosis

Ferroptosis involves three major mechanisms (Fig. 1): the occurrence mechanism, the defense mechanism, and the ferroptosis regulatory network [6]. The occurrence mechanism, considered the core pathway, is driven by the Fenton reaction, where ferrous iron (Fe^2 +^) and hydrogen peroxide (H₂O₂) generate reactive oxygen species (ROS) that attack polyunsaturated fatty acid phospholipids (PUFA-PLs) in cellular membrane, causing overwhelming lipid peroxide accumulation [3, 7]. The defense mechanism primarily revolves around the glutathione (GSH)-glutathione peroxidase 4 (GPX4) pathway, which detoxifies lipid peroxides, supported by system Xc^−^ -mediated cystine uptake [8]. Additionally, ferroptosis suppressor protein 1 (FSP1) and guanosine triphosphate cyclohydrolase I (GCH1) also contribute to ferroptosis inhibition [9]. The ferroptosis regulatory network extends beyond these core and defense pathways and comprises various enzymes, proteins, pathways and even organelles that could modulate the core or defenses mechanisms directly or indirectly, such as nicotinamide adenine dinucleotide phosphate (NADPH) [10], nuclear factor erythroid 2-related factor 2 (NRF2) [11]and Kelch-like ECH-associated protein 1 (KEAP1) [12]. The growing list of ferroptosis-related molecules underscores its complexity and therapeutic potential.

Despite advances in hematologic cancer therapies, mechanisms of disease progression and drug resistance remain incompletely understood, posing ongoing challenges in management. Recent studies have highlighted the critical role of ferroptosis in cancer progression and treatment response [13], with both experimental compounds and approved drugs showing potential to exploit ferroptotic vulnerability and overcome resistance [14, 15]. Unlike solid tumors, hematologic malignancies represent non-solid cancers—including leukemias, lymphomas, and multiple myeloma—that are characterized by dispersed tumor cell populations lacking a fixed tissue architecture. These malignancies exhibit marked cellular heterogeneity, dynamic clonal evolution, and a strong dependence on the bone marrow microenvironment. Importantly, they display distinctive metabolic features, particularly in in iron and lipid metabolism [16–18]. Such metabolic dependencies lead to elevated basal oxidative stress and disrupted redox homeostasis, making hematologic cancer cells inherently more vulnerable to ferroptosis than solid tumors. This heightened ferroptotic susceptibility offers unique opportunities to exploit redox and metabolic vulnerabilities as therapeutic targets in hematologic malignancies.

Given these characteristics, understanding ferroptosis mechanisms in hematologic malignancies offers novel insights into disease pathogenesis and opens potential therapeutic avenues [19]. This review focuses on the molecular regulation of ferroptosis and explores its applications in treating hematological malignancies.

## Mechanisms of ferroptosis

### The occurrence mechanisms of ferroptosis

#### Lipid peroxidation leads to ferroptosis

O_2_, PUFA-PLs and labile iron are three indispensable elements required for inducing ferroptosis. The overwhelming accumulation of lipid peroxides is a defining process and the final outcome of ferroptosis. During this process, lipid peroxyl radical formed by the reaction between molecular oxygen and PUFA-PLs initiate a self-propagating chain reaction, leading to irreversible lipid peroxidation [20]. Additionally, labile iron plays an important role in the subsequent auto-oxidation reaction of lipid peroxides by acting as the “driving force”, supplying an H-atom to the oxidative products for the next round of reaction [3].

PUFA-PLs, carrying one more carbon-carbon double bonds, are widely regarded as the target of oxidation in ferroptosis. The biosynthesis of PUFA-PLs is regulated by two key enzymes: Acyl-CoA synthetase long-chain family member 4 (ACSL4) and lysophosphatidylcholine acyl transferase 3 (LPCAT3), which can catalyze PUFAs into long-chain acyl-CoA and facilitate the attachment of phospholipids (PLs) to PUFAs [21]. The class of PLs includes phosphatidylethanolamine (PE), phosphatidylcholine (PC), and other diacyl PLs that combine with PUFAs to form PUFA-PLs. Ferroptosis-like death has been observed in cells containing various types of PUFA-PLs, especially those enriched with PUFA-PEs acylated with 20:4(an acyl chain with 20 carbons and 4 double bonds) or 22:4, due to their increased susceptibility to ROS [22]. Therefore, supplementation with arachidonic acid (AA), a kind of PUFA consisting of 20:4, increases the sensitivity of cancer cells to ferroptosis, whereas replacing PUFAs with monounsaturated fatty acids (MUFAs) mitigates lipid peroxidation, thereby protecting against ferroptosis [21, 23].

Hydroxyl radical (HO·), produced from Fenton reaction between H_2_O_2_ and Fe^2+^, are the most prevalent ROS that initiate lipid peroxidation. However, other ROS species, such as superoxide anion(O_2_·) and Hydroperoxyl radical(HOO·), derived from the mitochondrial electron transport chain (ETC) or NAD(P)H oxidase (NOX) enzymes, also contribute to lipid peroxidation, suggesting that the induction of ferroptosis is not dependent on specific hydroperoxides [21, 24]. ROS-initiated lipid peroxidation is the most direct and common oxidative way in ferroptosis. In addition, lipoxygenase (LOX) enzymes, a family of iron-containing dioxygenases, enzymatically catalyze lipid peroxidation [25]. Overexpression of LOX sensitizes cells to ferroptosis in certain diseases, whereas the inhibition of LOX activity provides protection against RSL3-induced ferroptosis [21]. Although LOXs contribute to the cellular pool of lipid hydroperoxides that promote lipid autoxidation, they are dispensable to the ferroptosis process, since lipid peroxidation can still be induced in the some human cell lines that do not express LOX proteins [25]. Moreover, 5-LOX, p12-LOX, and 15-LOX-1 have all been shown to increase sensitivity of ferroptosis, yet no single LOX is necessary to this process [26].

#### The role of iron in ferroptosis

Iron is a fundamental element in cellular metabolism, participating in numerous biochemical pathways in both its ferrous (Fe^2 +^) and ferric (Fe^3 +^) forms. These redox-active species are central to the initiation and execution of ferroptosis. Within the cell, a key source of this reactive iron is the labile iron pool (LIP) [27], a transient reservoir loosely bound to small molecules and proteins. The LIP's dynamic nature, partly maintained through interactions between the ferritin heavy chain (FTH) and ferritin light chain (FTL), contributes to spontaneous iron oxidation during ferroptosis [28]. The LIP is dynamically supplied through two primary pathways: cellular iron import and ferritin degradation. The principal route of iron uptake involves transferrin-mediated transport [29]. Transferrin binds to its receptor, transferrin receptor 1 (TfR1), on the cell surface, enabling the internalization of transferrin-bound iron via receptor-mediated endocytosis. Upregulation of TfR1 enhances iron influx, expands the LIP, and promotes lipid peroxidation—the hallmark of ferroptotic cell death [30]. This process is strictly dependent on iron bioavailability, as TfR1 exclusively recognizes iron-loaded transferrin. Consistent with this, iron-free apo-transferrin does not induce cell death, while iron chelators effectively inhibit ferroptosis. Thus, the cytotoxic potential of transferrin is determined by its iron-loading status [31], highlighting transferrin saturation as a key physiological and pathological modulator of ferroptosis sensitivity. A second major contributor to the LIP is ferritinophagy, the selective autophagic degradation of ferritin. [32]. This selective autophagy pathway is mediated by the cargo receptor NCOA4, which recognizes ferritin and targets it for lysosomal degradation, leading to the release of free iron and thus an increase of LIP [33]. This process amplifies ROS accumulation and precipitates ferroptotic cell death. Consequently, NCOA4-mediated ferritinophagy serves as a pivotal regulatory mechanism linking iron storage and ferroptosis. Beyond its storage and transport functions, iron also drives ferroptosis through iron-dependent enzymatic reactions, particularly via lipoxygenase (LOX)-mediated lipid peroxidation [21, 34]. While iron remains the principal catalyst of ferroptosis, recent studies suggest that copper can induce similar oxidative cell death processes through related mechanisms [35]. Together, these findings underscore that the regulatory role of metal ions in ferroptosis is highly context-dependent, varying across different cell types and physiological conditions.

### The defense mechanisms of ferroptosis

Although lipid peroxidation is the central feature of ferroptosis, cells possess intrinsic defense mechanisms to counteract oxidative damage. Thereby ferroptosis occurs at a basal rate, wherein the mechanisms of occurrence and defense mutually maintain a balance. Once this equilibrium is disrupted, the accumulation of lipid peroxides surpasses the scavenging capacity of the defense mechanisms, ultimately resulting in the onset of ferroptosis [36, 37]. Various enzymes and metabolites that mediate redox systems function as the inner defense mechanism to prevent uncontrollable lipid peroxidation. The GSH-GPX4 pathway is widely recognized as a key ferroptosis defense system for converting the lipid peroxides into non-toxic lipid alcohols. Additionally, FSP1, GCH1 and dihydroorotate dehydrogenase (DHODH) also contribute to ferroptosis defense by acting as radical trapping antioxidants [38–40].

#### The GSH-GPX4 system

The GPX4 pathway is the primary defense mechanism against ferroptosis. GPX4 utilizes GSH as a hydrogen atom donor to reduce lipid peroxides into non-toxic lipid alcohols and simultaneously oxidize GSH to oxidized glutathione (GSSG) [41]. The system Xc^-^, composed of solute carrier family 3 member 2 (SLC3A2) and solute carrier family 7 member 11 (SLC7A11), facilitates cystine uptake for GSH biosynthesis [42]. Inhibition of the Xc^-^ system decreases intracellular GSH and promotes ferroptosis positively, similar to the effect of the ferroptosis inducer erastin [3, 43]. In addition to erastin, inhibitors of GPX4, such as RSL3, FIN56, and ML210, can directly target GPX4, leading to an overwhelming accumulation of lipid peroxidation and triggering ferroptosis (Table 1) [48, 53, 54].

**Table1:** The common inducers and inhibitors of ferroptosis

	Mechanisms	Drugs	Ref.
Inducer	Inhibits system Xc^-^	Erastin	[3]
		Sulfasalazine	[44]
		Sorafenib	[8]
	Inhibits GPX4	RSL3	[3]
		ML162	[45]
		ML210	[46]
		FIN56	[47]
		FINO2	[48]
Inhibitor	Inhibit accumulation of iron	Deferoxamine	[49]
	Inhibit lipid peroxidation	Ferrostatin-1	[50]
		Liproxstatin-1	[51]
		Vitamin E	[52]

#### The radical trapping antioxidants

The radical trapping antioxidants (RTAs) serve as secondary defense molecules against ferroptosis by neutralizing lipid peroxyl radicals [9]. The most active form of vitamin E, α-Tocopherol (α-TOH), has been shown to inhibit ferroptosis through scavenging lipid peroxides in vitro [25]. Additionally, FSP1, first identified in 2002 [55, 56], enhances the production of reduced coenzyme Q10 (CoQ10), which acts as a lipid-soluble antioxidant [41, 57]. This ferroptosis defense mechanism seems to be independent of GSH-GPX4 system, because the FSP1 knockout does not affect GSH levels [41]. Other ferroptosis inhibitors, such as GCH1 facilitate the synthesis of another RTA—tetrahydrobiopterin (BH4)—and prevents the peroxidation of phosphatidylcholine containing two PUFA chains [39, 41, 58]. BH4 is also regulated by dihydrofolate reductase (DHFR), which regenerates oxidized BH4 [40]. However, the roles of RTA-related ferroptosis inhibitors are not uniform across different cell types, suggesting that the interaction of these antioxidants with ferroptosis may vary depending on the cellular context.

### The regulatory network of ferroptosis

**Figure 2:**
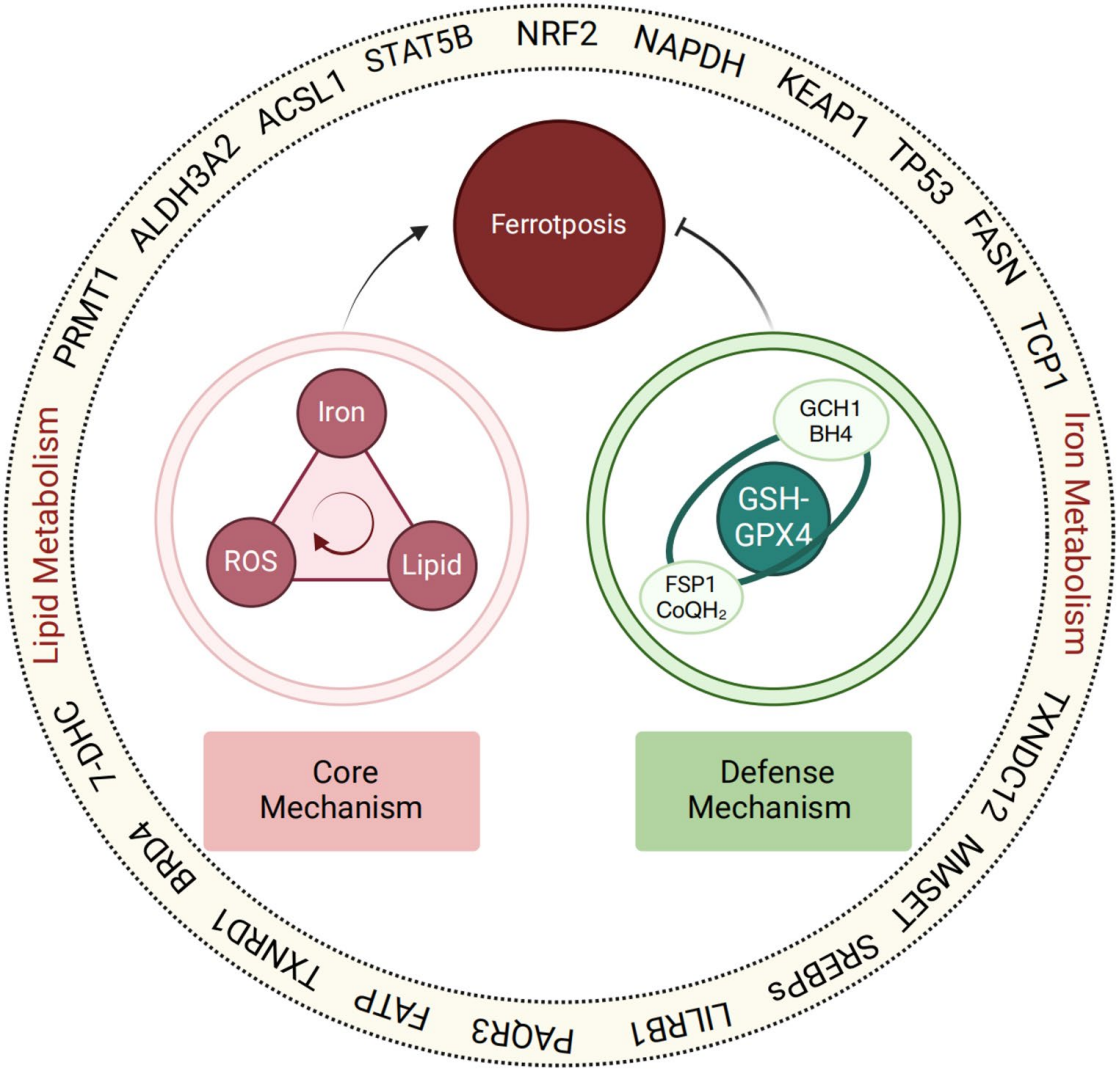
Ferroptosis regulatory network. Ferroptosis is multidimensional in regulatory mechanisms involving multiple biological entities, regulatory network compose numerous enzymes, proteins, and transcription factors are capable of significantly influencing the sensitivity of ferroptosis through intertact with core mechanism and defense mechanism

Molecules with that regulate the core mechanisms or defense mechanisms directly or indirectly can be grouped within the ferroptosis regulatory network (Fig. 2). This network consists of regulators that interact with, but are distinct from, the core and defense mechanisms. Recent research into ferroptosis has revealed a growing number of enzymes, proteins, transcription factors, and even multiple metabolic reactions that involved in lipid synthesis and peroxidation, iron metabolism, and redox reaction. [31, 40, 59–62]. However, the impact of each factor on ferroptosis can vary considerably depending on cell type and physiological state [40].

Cellular metabolism plays a central role in determining ferroptosis sensitivity. Key metabolic processes such as glutamine metabolism [31], glucose metabolism [62], and mitochondrial metabolism [24] converge to regulate this form of cell death. During glutamine metabolism, also known as glutaminolysis, L-glutamine is converted to α-ketoglutarate, fueling the tricarboxylic acid (TCA) cycle and linking mitochondrial activity to cysteine-deprivation–induced ferroptosis [31, 63]. Glucose metabolism similarly influences ferroptotic responses through glycolysis, the pentose phosphate pathway(PPP), and glycogenesis. Under glucose-deprived conditions, activation of AMP-activated protein kinase (AMPK) suppresses ferroptosis by inhibiting lipid biosynthesis [62]. In contrast, glucose-6-phosphate dehydrogenase (G6PD), a key enzyme in the PPP, supports cancer cell survival by blocking ferroptosis [64]. It does so through downregulation of cytochrome P450 oxidoreductase and modulation of the AMPK–mTOR pathway, maintaining redox balance and promoting proliferation [64, 65]. The PPP also produces NADPH, which can either inhibit or enhance ferroptosis depending on cellular redox status [10].

Mitochondria integrate these metabolic cues to shape ferroptotic vulnerability. As a major source of ROS, mitochondrial respiration and electron transport amplify lipid peroxidation and ferroptosis [62, 66]. Interestingly, ferroptotic outcomes depend on the cell’s energy status—when ATP is depleted, AMPK activation suppresses ferroptosis, whereas sufficient ATP levels permit ferroptotic execution [62, 66]. Thus, mitochondrial bioenergetics, biosynthesis, and ROS production collectively define the metabolic landscape that governs ferroptosis [24].

Multiple cellular molecules participate in the ferroptosis regulatory network. Among these regulators, NRF2 stands out as a key transcription factor governing ferroptosis through the control of both iron metabolism and lipid peroxidation [67]. NRF2 maintains iron homeostasis by regulating the expression of HECT and RLD domain-containing E3 ubiquitin protein ligase 2 (HERC2) and vesicle-associated membrane protein 8 (VAMP8) [11]. Loss of NRF2 reduces HERC2 and VAMP8 levels, leading to ferritin accumulation and impaired ferritinophagy [11]. This disruption increases the labile iron pool (LIP) and heightens susceptibility to ferroptosis. Additionally, NRF2 mitigates lipid peroxidation by upregulating antioxidant and glutathione-related genes, including glutamate–cysteine ligase (GCLC/GCLM), glutathione synthetase (GSS), and SLC7A11 [68, 69].

In summary, ferroptosis is governed by an intricate regulatory network involving diverse metabolic pathways and signaling molecules. These interconnected systems collectively determine ferroptotic susceptibility and highlight the context-dependent nature of this unique form of cell death.

## Role of ferroptosis in hematological malignancies

Hematological malignancies exhibit diverse pathogenic mechanisms, yet they share common characteristics, including rapid proliferation, impaired differentiation and maturation, and immune evasion. These traits make them key research tragets and potential therapeutic opportunities. To maintain the high proliferative and differentiated state, hematological malignancies evolve specific metabolic adaptations and molecular modifications that enhance resistance to cell death, especially ferroptosis. Ferroptosis is regulated by multiple pathways, and hematological malignancy cells appear to evade ferroptotic death by modifying certain key mechanisms. Nevertheless, the precise ways these cells employ to bypass ferroptosis remain incompletely understood, presenting a promising area to exploit ferroptosis as a therapeutic approach. Further investigation into its role in hematological malignancies could provide new insights into both pathogenesis and treatment strategies.

### Leukemia

Leukemia is a group of bone marrow-derived non-solid tumors characterized by an excessive accumulation of immature myeloid or lymphatic cells in an abnormal state of differentiation and proliferation. To sustain their uncontrolled proliferation, leukemia cells exhibit high metabolic activity and elevated ATP demand, which in turn generates excessive ROS. This oxidative stress environment may influence ferroptosis sensitivity.

#### AML

##### Core mechanisms and related regulatory network of ferroptosis in AML

Lipid composition and metabolism are essential for the acute myeloid leukemia (AML). Compared to normal hematopoietic cells, AML cells display elevated levels of multiple free fatty acids, reflecting enhanced lipid biosynthetic activity [70]. The transcription factor CCAAT-enhancer binding protein α (C/EBPα) contributes to the susceptibility to lipid oxidative stress-induced ferroptosis in Fms-like tyrosine kinase 3 (FLT3)-mutant AML through intervening fatty acid biosynthesis [71]. C/EBPα, essential for both normal and leukemic differentiation, promotes fatty acid synthesis and desaturation via the fatty acid synthase (FASN)–stearoyl-CoA desaturase (SCD) axis [71]. In *FLT3*-mutant AML, C/EBPα cooperates with FLT3 signaling to boost lipid anabolism, whereas loss of either C/EBPα or FLT3 reduces MUFA incorporation into membrane phospholipids by suppressing SCD expression. This shift enhances lipid peroxidation and sensitizes cells to ferroptotic stress. Thus, C/EBPα protects *FLT3*-mutant AML cells from ferroptosis, and its inhibition increases ferroptotic vulnerability following FLT3 blockade [71]. Another key regulator, basic helix-loop-helix ARNT like 1(Bmal1), is overexpressed in AML and associated with poor prognosis [72]. Bmal1 promotes AML progression through ferroptosis suppression by facilitating enhancer of zeste 2 polycomb repressive complex 2 subunit(EZH2)-mediated methylation of EBF transcription factor 3(EBF3) promoter and downregulating EBF3 and ALOX15, which is required for lipid peroxidation that contributes to ferroptosis.

Disrupted iron homeostasis is also implicated in AML pathogenesis, which plays a significant role in various stages of myeloid differentiation and proliferation [73, 74]. Key iron-regulatory genes—many of which also govern ferroptosis—show altered expression in AML. Leukemic blasts exhibit elevated expression of iron-regulatory genes such as TFR1 and TF genes, indicating elevated intracellular iron fluxes compared to healthy hematopoietic progenitors [74]. FTH and FTL, crucial for maintaining the LIP, are overexpressed in AML, thereby inhibiting ferroptosis [75, 76]. FTH1 overexpression, in particular, has been associated with reduced chemosensitivity through activation of the NF-κB pathway [77], whereas FTH1 deletion extends survival in AML mouse models [78]. These findings underscore that iron homeostasis genes critically influence AML progression and treatment response, though their precise mechanistic interplay with ferroptosis remains to be fully elucidated.

Oxidative stress also plays a key role in AML progression and ferroptosis regulation. Thioredoxin domain–containing protein 12 (TXNDC12), an endoplasmic reticulum (ER) chaperone that maintains protein folding homeostasis, is upregulated in human AML cell lines (HL-60 and K562). TXNDC12 suppresses lipid peroxidation and ferroptosis through a GPX4-independent antioxidative mechanism, and its inhibition enhances ferroptosis-mediated AML suppression [79]. Additionally, high mobility group box 1 (HMGB1) plays an important role in leukemia pathogenesis and chemotherapy resistance. A specialized AML cell line, HL-60 cell line expressing NRAS^Q61^, exhibited increased sensitivity to chemotherapeutic agents following erastin treatment [80]. In the NRAS^Q61^ HL-60 cell line, erastin treatment triggers HMGB1 translocation from the nucleus to the cytoplasm, promoting ferroptotic cell death [81]. Conversely, HMGB1 knockdown reduces erastin-induced ROS generation and ferroptosis through an iron-mediated lysosomal pathway, highlighting its potential as a therapeutic target in AML.

##### Defense mechanisms and regulatory network of ferroptosis in AML

AML cells employ multiple defense mechanisms to evade ferroptotic cell death, primarily by enhancing antioxidant pathways and modulating iron and lipid metabolism. In patient bone marrow samples, both NRF2 and GPX4 are significantly upregulated compared with healthy controls [82]. NRF2 overexpression increases GPX4 levels, and elevated GPX4 correlates with poor prognosis, suggesting that the NRF2–GPX4 axis constitutes a critical ferroptosis evasion strategy in AML [82]. Consistent with this, GPX4 inhibition induces ferroptosis in AML cells both in vitro and in vivo, evidenced by characteristic mitochondrial lipid peroxidation, highlighting GPX4 as a potential therapeutic target [83].

Beyond GPX4, other molecules contribute to ferroptosis regulation in AML. Aldehyde dehydrogenase 3A2 (ALDH3A2), an enzyme that detoxifies long-chain fatty aldehydes and generates C16–C18 fatty acids, is essential for AML cell survival under specific metabolic conditions. ALDH3A2 prevents oxidative damage by oxidizing aliphatic aldehydes, and its depletion induces ferroptosis, acting synergistically with GPX4 inhibition [84]. However, how alteration of this redox state and lipid metabolism affect ferroptotic process in AML still needs further research. The redox state in tumor microenvironment also modulates ferroptosis in AML. Lu et al. [85] reported that M2 macrophages in AML secrete high levels of growth differentiation factor 15 (GDF-15) enhances the expression of the SLC7A11/GPX4 axis, thereby inhibiting ferroptosis and contributing to resistance of mitoxantrone (MTX) in AML. Similarly, CCL20 from M2 macrophages promotes iron homeostasis in AML cells by increasing SLC7A11 activity, mitigating mitochondrial damage, and reducing ferroptotic sensitivity, thereby facilitating chemotherapy resistance [86].

FSP1 deficiency significantly inhibit AML progression and this process is mediated by the principal enzyme responsible for m^5^C deposition in RNA—NOP2/Sun RNA methyltransferase 2 (NSUN2) [87]. NSUN2 is significantly overexpressed in AML patients, mechanistically, NSUN2 mediates m^5^ C methylation in the 3’UTR of FSP1 mRNA, enabling YBX1 binding to stabilize the transcript and maintain FSP1 protein levels and protecting AML cells from ferroptotic stress by suppressing lipid peroxidation [87]. Furthermore, Zhou et al. [88] identified protein arginine methyltransferase 1 (PRMT1) also functions as a ferroptosis suppressor by downregulating ACSL1, a key driver of lipid peroxidation, with PRMT1 loss increasing ferroptotic susceptibility. In addition, the long non-coding RNA LINC00618 is frequently downregulated in AML; it promotes ferroptosis by suppressing SLC7A11 expression via inhibition of LSH-mediated transcriptional activation [89].

#### ALL

Research on ferroptosis in acute lymphoblastic leukemia (ALL) remains limited, but emerging studies suggest that ALL cells display unique ferroptotic vulnerabilities. Lanlode et al. [90] performed whole-genome CRISPR/Cas drop-out screens on seven B-cell ALL cell lines, identifying a set of genes representing unique functional vulnerabilities in B-ALL. Enrichment analysis revealed that several of these genes are involved in in pathways related to ferroptosis induction and defense, including selenocompounds, lipids, mevalonate, GSH and iron metabolism. Notably, ferroptosis suppressor FSP1 exhibits low mRNA and protein levels in B-ALL cells, and restoring FSP1 expression increases resistance to ferroptosis, suggesting that low FSP1 levels in B-ALL may contribute to the ferroptosis sensitivity [90]. Simlialy, Pontel et al. [91] reported FSP1-epigenetic silencing in both *T*- and B-ALL, in which the promoter of the gene coding for FSP1 is hypermethylated, silencing the expression of *FSP1* and creating a selective dependency on GSH-centered anti-ferroptosis defenses, this metabolic vulnerability that might be of therapeutic interest in ALL.

The tumor suppressor PAQR3 is frequently downregulated in ALL and can inhibit proliferation and induce apoptosis in leukemic cells [92]. Jin et al. [92] demonstrated that PAQR3 could exacerbate ferroptosis in ALL, and this cell death could be rescued by NRF2 overexpression, highlighting the interplay between pro-ferroptotic signals and antioxidant defenses. In addition, Tian et al. [93] identified five ferroptosis-associated hub genes in T-cell ALL through a protein-protein interaction (PPI) network, and found that Lipocalin2(LCN2) was highly expressed in T-ALL, and knockdown of LCN2 could enhance RSL3-induced ferroptosis, characterized by increased iron, malondialdehyde (MDA), COX2 levels and decrease of GSH and GPX4 levels.

#### CML and CLL

Ferroptosis is relatively underexplored in CML and chronic lymphoblastic leukemia (CLL). Even though several ferroptosis-related biomarkers and prognostic scores have been identified through bioinformatics analyses [94–96], comprehensive molecular studies investigating the role of ferroptosis in CML and CLL are limited.

Thioredoxin reductase1(TXNRD1) acts as a key ferroptosis suppressor in CML, and its inhibition induces ferroptosis in cysteine-deplete CML cells [97]. Notably, cysteine depletion alone did not induce lipid peroxidation in K562 cells, suggesting that that CML cells rely on coordinated regulation of multiple antioxidant defense systems, including TXNRD1 and GSH/GPX4, to resist ferroptosis [97]. Additional defense mechanisms involve transcription factors such as GATA-1, which protects CML cells by upregulating GPX4, thereby preventing lipid peroxidation and ferroptotic death [98]. Moreover, Peng et al. [99] reported that enolase 1 (ENO1) serves as a key biomarker for CML response to tyrosine kinase inhibitors (TKIs), ENO1 inhibiton could augment TKIs sensitivity and promote the ferroptosis susceptibility in TKIs-resistant cells by ultimately inducing GPX4 autophagic degradation through AMPK/mTOR. In CLL, Nardi et al. [100] identified that apolipoprotein E (ApoE) as a serum protein capable of suppressing CLL cell proliferation through a ferroptosis-related mechanism modulated by intracellular copper levels. Although CLL cells activate transcriptional programs to counteract ApoE toxicity, they remain vulnerable to pharmacologically induced ferroptosis, indicating that induction of ferroptosis could be a promising strategy to overcome CLL's resistance to apoptotic cell death and Bcl-2 inhibitors [100].

### Lymphoma

Lymphoma, the sixth most common cancer worldwide, comprises over 90 subtypes arising from the clonal proliferation of various subsets of lymphocytes [101]. Disruptions in lipid metabolism, iron homeostasis and oxidative damage contribute to lymphoma progression and drug resistance [102–104], suggesting a potential role for ferroptosis in both disease progression and treatment. However, studies on ferroptosis in lymphoma remains limited, with only a few identified regulatory molecules. Given the complexity and diversity of lymphoma subtypes, this section presents a general overview of existing research on ferroptosis related to lymphoma.

#### Core mechanisms and regulatory network of ferroptosis in Lymphoma

Abnormalities in lipid and amino acid metabolism have been observed in lymphoma cells, which is involved in ferroptosis. The ALOX5 gene, encoding arachidonate 5-lipoxygenase, is highly expressed in approximately 50% patients with germinal center B cell (GCB)-like diffuse large B-cell lymphoma (DLBCL), correlating with susceptibility to ferroptosis [105]. Wang et al. [106] utilized untargeted metabolomics analysis to investigate serum samples from 60 newly diagnosed DLBCL patients, and revealed that glutamine metabolism is up-regulated in DLBCL patients, characterized by elevated glutamine and decreased α-ketoglutarate (α-KG) derivatives. One of α-KG derivatives—Dimethyl α-ketoglutarate (DM-αKG) could be converted to 2-hydroxyglutarate by malate dehydrogenase 1, leading to ROS production in double-hit lymphoma (DHL) cells, then, high levels of ROS contributed to ferroptosis induction by promoting lipid peroxidation and TP53 activation. What’s more, this process can be reduced by a ferroptosis inhibitor Ferrostatin-1 (Fer-1). Therefore, α-KG suppresses DHL cells growth as a ferroptosis promoter, and the decreased α-KG may reflect the ferroptosis escape resulting in enhanced DLBCL survival. Targeting α-KG may be considered as a new promising therapeutic strategy for DHL patients. Additionally, tailless complex polypeptide 1 (TCP1), a subunit of chaperonin-containing T-complex protein-1 (CCT), has been identified as a positive regulator of ferroptosis in GCB-DLBCL. TCP1 binds to ACSL4, reducing its ubiquitination and degradation, thereby increasing the accumulation of PUFA-PLs on the cell membrane and subsequently promoting ferroptosis [107]. 

#### Defense mechanisms and regulatory network of ferroptosis in lymphoma

The defense mechanism of ferroptosis play a key role in progression and prognosis in lymphoma as well. GPX4 is overexpressed in DLBCL compared to non-neoplastic B cells [108]. Immunohistochemical analyses have classified DLBCL patients into GPX4-positive and GPX4-negative groups, revealing that GPX4 positivity correlates with significantly poorer overall (OS) and progression-free survival (PFS). GPX4 overexpression was also identified as an independent prognostic predictor, though the mechanisms driving these clinical associations require further investigation [108]. Bromodomain-containing protein 4(BRD4), a member of the mammalian bromodomain and extra-terminal domain (BET) protein family, plays a central role in protecting DLBCL cells from lipid peroxidation [109]. BRD4 functions as a platform for various transcriptional regulators, and connects super-enhancer with promoter regions, thereby stabilizing the transcriptional machinery [110]. Several ferroptosis suppressor genes, such as SLC7A11 and FSP1, are downregulated following BRD4 inhibition in GCB-DLBCL cells. Inhibition or knockdown of BRD4 reduces FSP1 levels, thereby heightening ferroptotic vulnerability in germinal center B-cell (GCB)-type DLBCL. Collectively, these findings suggest that BRD4 is an important contributor in ferroptosis suppression in GCB-DLBCL. Progesterone and adiponectin receptor 3 (PAQR3), a known tumor suppressor, is downregulated in DLBCL, and its low expression is associated with the poor prognosis [111].

PAQR3 inhibits DLBCL progression in a ferroptotic way by restraining low-density lipoprotein receptor (LDLR) expression, thereby increasing GSH level and promote the expression of GPX4 and SLC7A11. FASN is another key player implicated in poor prognosis and drug resistance in DLBCL, Zhong et al. [112] reported that FASN is markedly elevated in DLBCL patients with worse prognoses, particularly those adriamycin (ADM) chemotherapy. FASN overexpression reduces both total and lipid ROS levels, increases GPX4 expression, and decreases the lipid peroxidation marker 4-hydroxynonenal (4-HNE). Conversely, FASN knockdown enhances the ferroptosis-promoting effect of ADM, which could be reversed by Fer-1 treatment, illustrating the anti-ferroptosis effect and pro-tumor function of FASN in DLBCL. Furthermore, in more aggressive DLBCL cases, lymphoma cells utilized FASN as a tumor escape tool to enhance their survival prospects. Mechanistically, overexpression of FASN inhibites ferroptosis by activating the NF-κB/STAT3 signaling pathway, thereby promoting GPX4 expression [112].

The EZH2 inhibitors(EZH2i) have shown promising effects in various tumors in clinic [113], but serious primary drug resistance limites their clinical application [114]. Yu et al. [115] showed that EZH2 inhibition increases intracellular iron through upregulation of TfR1, ultimately led to EZH2i resistance and treatment failure in DLBCL cells. This process involves elevated heat shock protein family A member 5 (HSPA5) expression, which stabilizes GPX4 by reinforcing the HSPA5–GPX4 interaction, thereby strengthening ferroptosis resistance. Notably, the combination of erastin with EZH2i could reverse the upregulation of GPX4, synergistically inducing ferroptosis and inhibiting EZH2i-resistant DLBCL cell growth in vitro and in vivo. These findings highlight iron-dependent ferroptosis resistance as a mechanism of drug failure and point to ferroptosis induction as a promising combination strategy [115].

In Burkitt lymphoma (BL), 7-dehydrocholesterol reductase (DHCR7) and its substrate 7-dehydrocholesterol (7-DHC) have been implicated in ferroptosis regulation [103]. DHCR7 catalyzes the final step in cholesterol biosynthesis and its inhibition leads to 7-DHC accumulation, which protects lipid from autoxidation due to its superior reactivity towards peroxyl radicals. DHCR7 mutations were observed in approximately 9.8% of BL cases [116], with certain variants (T93M, N274K, L306R, E448K, and V353fs) found to be dysfunctional, failing to metabolize 7-DHC. This dysfunction results in 7-DHC accumulation, shifting BL cells toward ferroptosis resistance and a more aggressive phenotype [103]. Signal transducer and activator of transcription 5B(STAT5B) has been identified as a positive regulator of ferroptosis in mantle cell lymphoma (MCL), which is upregulated and acts as a positive regulator of ferroptosis escape in MCL [117, 118]. It enhances the transcription of DCAF13, which promotes p53 ubiquitination and degradation, leading to increased expression of SLC7A11 and GPX4. Consequently, this process diminishes ferroptosis in MCL and contributes to the disease progression, underscoring STAT5B as a potential target to restore ferroptosis sensitivity in MCL [117].

Despite the emerging evidence linking ferroptosis to lymphoma pathogenesis and treatment response, current studies remain relatively fragmented and mechanistically superficial compared with leukemia. Most findings are limited to in vitro observations or specific lymphoma subtypes such as DLBCL, while comprehensive analyses across other entities, including Hodgkin and T-cell lymphomas, are scarce. Furthermore, the interactions between ferroptosis and the tumor immune microenvironment, metabolic reprogramming, and drug resistance remain largely unexplored. Future research should aim to delineate subtype-specific ferroptosis regulatory networks and evaluate the translational potential of ferroptosis modulators in preclinical lymphoma models.

### Myeloma

Multiple myeloma (MM) is the second most common hematologic malignancy, characterized by the accumulation of monoclonal plasma cells in the bone marrow. Despite advances in therapy improving overall survival, high recurrence rates remain a major challenge. Recent studies suggest ferroptosis plays a role in MM pathogenesis, offering potential therapeutic insights.

#### Core mechanisms and regulatory network of ferroptosis in MM

MM cells exhibit abnormal iron metabolism, contributing to increased cell proliferation and drug resistance [17, 119]. This dysfunction is accompanied by elevated serum iron levels and abnormal expression of several iron-related genes, such as TFCR and SLC40A1 [120]. Camiolo et al. [17] reported that iron significantly impacts both plasma cells and the TME in MM.

Iron trafficking not only modulates interactions between MM cells and macrophages but also contributes to the development of bortezomib (BTZ) resistance. However, the precise role of ferroptosis in these iron-dependent mechanisms requires further elucidation.In addition to iron dysregulation,aberrant lipids synthesis and metabolism support MM cells proliferation and abnormal differentiation. ACSL4 is crucial in ferroptosis, serving as a key molecule in the synthesis of PUFA-PLs. Zhang et al. [121] reported ACSL4 is overexpressed in MM cell lines and MM patients samples. Knockdown of ACSL4 reduced sensitivity to RSL3-induced ferroptosis, underscoring its role as a ferroptosis-related vulnerability in MM. The cytogenetic abnormality t(4;14), associated with poor clinical outcomes, leads to overexpression of the multiple myeloma SET domain-containing protein (MMSET) [122, 123]. Zhang et al. [124] reported that MMSET enhances ACSL4 transcription by binding to its promoter region, increasing PUFA levels and ferroptosis sensitivity in t(4;14)-positive MM cells. This finding suggests that MMSET not only drives oncogenic progression but also modulates ferroptotic susceptibility through lipid metabolic reprogramming.

The abnormal lipid composition and metabolism within the TME influence the ferroptosis of MM cells. Leukocyte immunoglobulin-like receptor B1 (LILRB1), associated with poor MM prognosis, maintains cholesterol metabolic homeostasis to protect MM cells from ferroptosis [125]. Mechanistically, LILRB1 forms a complex with the low-density lipoprotein receptor and LDLR adapter protein 1 to protects MM cells from ferroptotic cell death via facilitating the uptake of LDL/cholesterol. Clinically, MM patients with hypercholesterolemia exhibited reduced ferroptosis activation compared to those with normal cholesterol levels, further highlighting cholesterol’s protective role in MM ferroptosis resistance. Moreover,bone marrow stromal cells (BMSCs) promote MM cell proliferation by differentiating into adipocytes and releasing free fatty acids [18]. The lipidomic analyses of bone marrow aspirates revealed significantly decreased arachidonic acid (AA) levels in MM patients. Low AA doses enhance MM cell viability, whereas high AA doses induce ferroptosis by reducing GPX4 expression, an effect reversed by Fer-1 [18]. These findings indicate that MM cells may promote self-proliferation and escape from ferroptosis by inhibiting excess AA uptake. Not only the fatty acids, components of sterol lipids are also modified by BMSCs. BMSC interactions increased lanosterol production in MM cells [126], which triggers ROS accumulation and sensitizes MM cells to ferroptosis [126].

#### Defense mechanisms and regulatory network of ferroptosis in MM

The ferroptosis defense mechanism in MM cells exists abnormality as well. SLC7A11, a key molecule in ferroptosis defense mechanism, is highly expressed in patients with MM, and correlates with disease progression and poor prognosis [127]. Patients with elevated SLC7A11 expression show increased sensitivity to erastin-induced ferroptosis, whereas SLC7A11 knockdown significantly inhibits MM proliferation and induces ferroptotic cell death. Further investigation into its regulatory mechanisms may provide valuable insights for improving MM treatment [127]. Moreover,Ma et al. [128] reported that MM patients with prolonged survival exhibited lower CD36 expression on CD8+ T cells compared to those with shorter survival. CD36 mediates fatty acids uptake,which induces lipid peroxidation and ferroptosis in tumor-infiltrating CD8+ T cells. CD36 deficiency reduces oxidative stress and enhances cytotoxic cytokine production, thereby increasing antitumor activity. Ferroptosis-related signaling pathways and genes are also implicated in MM tumorigenesis and prognosis.These pathways may refine prognostic prediction, improve the ISS/RISS staging systems, and inform the design of ferroptosis-based immunotherapies [129–132].

Additionally, Jiang et al. [133] demonstrated that BMSCs could prevent MM cells from spontaneous ferroptosis in MM cells through direct cell–cell interactions.This process involves activation of the CD40/CD40L signaling pathway, which increases the expression of SUMO-specific protease 3 (SENP3) in MM cells. SENP3 de-conjugated SUMO2 at lysine 75 residue to stabilize GPX4 protein, thereby consuming ROS to obviate ferroptosis in MM cells. A long non-coding RNA FEZF1 antisense RNA 1 (FEZF1-AS), highly expressed in MM, also inhibited ferroptosis via Vir-like m6A methyltransferase associated protein (KIAA1429)-mediated N6-methyladenosine (m6A) modification [134]. In this process, FEZF1–AS1 enhanced the stability and expression of KIAA1429 mRNA, KIAA1429, in turn, promotes m6A modification of OTUB1 mRNA, facilitating its translation via YTHDF1. OTUB1 then stabilized SLC7A11 expression by deubiquitination, thereby reinforcing ferroptosis resistance in MM cells.

Although several studies have begun to uncover ferroptosis-related mechanisms in multiple myeloma, particularly involving iron metabolism, lipid homeostasis, and stromal interactions, the overall understanding remains limited. The molecular determinants that dictate ferroptosis sensitivity in genetically distinct MM subtypes and the interplay between ferroptosis and drug resistance pathways are still poorly defined. In addition, in vivo and clinical data validating ferroptosis as a therapeutic target in MM are lacking. Future investigations integrating multi-omics profiling, microenvironmental modeling, and pharmacologic screening are warranted to clarify the ferroptosis regulatory landscape and guide the rational design of ferroptosis-based therapeutic strategies in MM.

## Potential applications of ferroptosis-inducing therapies in hematological malignancies

In recent years, increasing studies highlight the essential anti-cancer role of ferroptosis inducers across various tumors, including hematological malignancies [135–137]. These agents not only exert direct cytotoxic effects but also enhance the efficacy of conventional treatments [138]. Furthermore, ferroptosis induction presents a promising strategy for overcoming drug resistance in cancer treatment [139, 140]. Here, we summarize key ferroptosis-inducing compounds against hematological malignancies based on preclinical studies and clinical trials (Table 2).

**Table2.** Ferroptosis inducers in hematological malignancies therapy

Disease	Cell lines	Drug	Target	Mechanism	Ref.
AML	HL60	Dihydroartemisinin (DHA)	Iron	Accelerated the degradation of ferritin, increased the LIP, increased intracellular iron release, and cellular ROS accumulation	[141]
	HL60	Perillaldehyde	GPX4	Decreased GPX4 protein expression, and depleted intracellular GSH	[142]
	Kas-1, HL60, NB4 and K562	Typhaneoside (TYP)	Iron	Ferritin degradation and ROS accumulation	[143]
	THP-1 and MOLM-13	Propionate	MT	Enhanced mitophagy	[144]
	NB4	Imetelstat	PUFAs	Increased levels of phospholipids containing PUFAs	[145]
	NB4 and HEL	PRMT1 inhibitor	ACSL1	Upregulated ACSL1 expression and increased the conversion of free fatty acids to fatty acyl-CoAs	[88]
	HL60	Neratinib	GPX4	Decreased GPX4 protein expression	[146]
	NPM1-mutant primary cell	Sulfasalazine	SLA7A11	Inhibited SLA7A11 and prevented GSH synthesis	[147]
	TP53-mutant AML patients	Eprenetapopt (APR-246)*	TXNRD1	Inhibited TXNRD1 and depleted GSH	[148–150]
	HL-60	Baicalein (BC)	SLC7A11/GSH/GPX4	Inhibited SLA7A11	[151]
CML	K562 and Meg-01	Hyperoside	NRF2/SLC7A11/GPX4	Inhibited NRF2 expression and suppressed SLC7A11/GPX4 axis	[152]
CML	K562 and Meg-01	Hyperoside	NRF2/SLC7A11/GPX4	Inhibited NRF2 expression and suppressed SLC7A11/GPX4 axis	[152]
T-ALL	JURKAT, MOLT-3, ALL-SIL and RPMI-8402	Poricoic acid A (PAA)	GSH	Reduced GSH levels and elevated lipid ROS	[153]
		nitric oxide donor-aurovertin B hybrids#	GPX4	Inhibited GPX4 through covalent binding to GPX4	[154]
DLBCL	U2932, and OCI-ly8	Artesunate (ART)	GPX4	Decreased GPX4 protein expression, and depleted GSH	[155]
	OCI-LY1	Rituximab	SLC7A11 and GPX4	The protein expression of SLC7A11 and GPX4 was decreased, and depleted GSH	[156]
	OCI-Ly1 and DoHH2	Combination of BTK inhibitors and BCL-2 inhibitors	NRF2, HMOX1 and GPX4	Downregulaed NRF2 and HMOX1, and GPX4 inactivation	[157]
	OCI-LY3, U-2932, DB and SUDHL-5	Ironomycin	Iron	Inducing ferritin degradation and ROS accumulation	[158]
	TP53-mutant primary cells	Eprenetapopt (APR-246)	TXNRD1	Depleted glutathione and inhibited TXNRD1	[159]
	OCI-LY1 and U2932	Kaempferitrin (KPF)	SLC7A11 and GPX4	The protein expression of SLC7A11 and GPX4 was decreased	[160]
NK/T cell lymphom	YT	Kayadiol	SLC7A11 and GPX4	Repressed SLC7A11 and decreased GPX4 activity	[159]
	Combination of decitabine and gemcitabine#	SLC7A11	Inhibited SLA7A11 in protein and mRNA levels	Combination of decitabine and gemcitabine#	[161]
MM	RPMI-8226 and U266	Andrographolide (Andro)	Nrf2, HO-1, and GPX4	Reduced Nrf2, HO-1 and GPX4 expression Oxidative injury accumulation	[162]
	RPMI-8226 and U266	Shikonin (SHK)	SLC7A11 and GPX4	Decreased SLC7A11 and GPX4 protein expression	[163]
	RPMI-8226 and U266	Eclipta prostrata (EEEP)	KEAP1/NRF2/HO-1	Increased KEAP1 expression and reduced NRF2 and HO-1 expression Decreased GSH and GPX4 levels	[164]
	RPMI-8226 and U266	Nitidine chloride (NC)	ABCB6/ACSL4/NRF2/HO-1	Increased ABCB6 protein degradation, and increased ferroptosis active markers(expression ACSL4, NRF2 and HO-1)	[165]
	RPMI-8226 and MM0.1S	Artesunate (ART)	SREBP2/ IPP/GPX4	Inhibited SREBP2 nuclear localization, decreased IIP and GPX4 level, ROS accumulation	[166]
	RPMI-8226 and MM0.1S	Fingolimod (FTY720)	PP2A/AMPK	Activated PP2A, dephosphorylated AMPK; downregulation of GPX4 and SLC7A11	[167]
	ARH-77, RPMI-8226, ARD, IM-9, U266	DCG066	Nrf2/HO-1	Reduced Nrf2 and HO-1 proteins expression, and decreased GSH	[168]
	RPMI-8226	*T*-5224	PI3K/AKT	Reduced PI3K and AKT phosphorylation; downregulation of GPX4 and SLC7A11	[169]

* agents in clinical development, others are in preclinical phase

### Leukemia

**Eprenetapopt (APR-246)**, a novel, first-in-class small molecule that restores wild-type p53 functions in TP53-mutant cells by facilitating their binding to DNA target sites, has entered clinical trials [170]. Mechanically, it increases oxidative stress by depletion of glutathione and inhibition of thioredoxin reductase, leading to ROS accumulation and further promoting tumor cell death [148, 149]. A Phase Ib/II study(ClinicalTrials.gov identifier: NCT03072043; Study Registration Dates: 2017–03-02) enrolled fifty-five TP53-mutant patients (40 myelodysplastic syndrome(MDS), 11 AML, and 4 MDS/myeloproliferative neoplasms(MPN)) to determine the safety, recommended phase II dose, and efficacy of eprenetapopt administered in combination with azacitidine [150]. The results showed that combination treatment is well tolerated, yielding high rates of clinical response and molecular remissions among patients with TP53-mutant MDS and oligoblastic AML. Whereas, APR-246 can also induce p53-independent cell death, Birsen and et al. [171] demonstrated that AML cell death occurring early after APR-246 exposure is suppressed by iron chelators, lipophilic antioxidants and inhibitors of lipid peroxidation, correlating with the accumulation of markers of lipid peroxidation, consequently, the cytotoxicity of APR-246 is exerted in a ferroptotic way. The ability of AML cells to detoxify lipid peroxides primes their sensitivity to APR-246 treatment.

Additional new synthesised compound, small-molecule inhibitors and repurposed drugs have shown ferroptosis-related antileukemic effects. **A nitric oxide donor with aurovertin B hybrids** suppresses GPX4 enzymatic activity and transcription by covalently binding, inducing ferroptosis in T-ALL [154]. **PRMT1 inhibitors** have been identified as ferroptosis regulators in AML [88]. PRMT1 inhibition or knockout leads to increased lipid peroxidation via upregulation of the ferroptosis-promoting enzyme ACSL1. **Imetelstat**, a first-in class telomerase inhibitor with clinical efficacy in hematological myeloid malignancies [172], induces ferroptosis in AML through fatty acid desaturase 2 (FADS2)-dependent lipid metabolic pathways [173]. Mechanically, imetelstat increases phospholipids desaturation, promoting lipid peroxidation in AML cells, and this enrichment of lipid desaturation could be diminished by FADS2 editing. Moreover, combining imetelstat with oxidative stress–inducing chemotherapy agents further amplifies its antileukemic efficacy [145]. **Neratinib**, a tyrosine kinase inhibitor approved by the food and drug administration (FDA) for the treatment of human epidermal growth factor receptor 2(HER2)-positive breast cancer, has been demonstrated to promote ferroptosis and inhibit brain metastases [174]. Ma and colleges demonstrated that neratinib induces ferroptosis in AML by increasing ROS, MDA, and Fe^2+^ levels while downregulating GPX4 and FTH1 expression [146]. **Sulfasalazine**, a clinically approved anti-inflammatory drug for inflammatory bowel disease [175], also induces ferroptosis in AML by inhibiting SLA7A11 and preventing GSH synthesis [176]. Notably, sulfasalazine enhances the efficacy of AML chemotherapy drugs, such as anthracyclines and erythromycin, leading to increased anti-leukemic activity [147].

Natural herbal extracts have recently gained increasing attention as potent ferroptosis-inducing agents in leukemia, owing to their diverse bioactive components and multi-targeted mechanisms. **Dihydroartemisinin (DHA)**, extracted from *Artemisia annua*, exhibits cytotoxic effects against various cancer types with subsequent generation of ROS and specific manifestations of ferroptosis. Du and colleagues demonstrated that DHA triggers ferroptosis in the HL-60 leukemic cell line by activiting of the AMPK/mTOR/p70S6k signaling axis and promoting iron release [141]. Similarly,Grignano, et al. [177]reported that DHA induces ferroptosis in variouse leukemic cells by triggering ferritinophagy, leading to intracellular iron release and lipid peroxidation via activating an early ferritinophagic flux. **Perillaldehyde**, a monoterpenoid isolated from *Ammodaucus leucotrichus Coss. & Dur*, induces lipid peroxidation in HL-60 promyelocytic leukemia cells by downregulating GPX4 and depleting intracellular glutathione. Importantly, perillaldehyde shows selective cytotoxicity against patient-derived AML cells while sparing normal hematopoietic cells, highlighting its promising therapeutic selectivity [142]. **Typhaneoside (TYP)**, a flavonoid from *Pollen Typhae*, induces ferroptosis in AML cells by promoting ferritin degradation and ROS accumulation while also influencing apoptosis and autophagy pathways [143]. **Baicalein (BC)**, a flavone compound with broad antitumor activity, triggered ferroptosis in AML via the SLC7A11/GSH/GPX4 pathway [151]. **Poricoic acid A (PAA)** a key constituent of *Poria cocos*, effectively inhibits T-ALL proliferation in vitro and in vivo by enhancing ROS production, depleting GSH, and increasing malondialdehyde (MDA) levels [153]. **Hyperoside**, a flavonol glycoside from *Hypericum and Crataegus species*, induces ferroptosis in CML cells through the NRF2/SLC7A11/GPX4 axis, confirmed by molecular docking and SLC7A11 overexpression [152].

Nanomedicine has further expanded the therapeutic potential of ferroptosis induction. Yu and colleagues developed a **glutathione (GSH)-responsive cysteine polymer-based ferroptosis-inducing nanomedicine (GCFN)**, serving as both an effective inducer of ferroptosis and a chemotherapeutic drug nanocarrier for AML treatment. GCFN depletes intracellular GSH and inhibits GPX4, resulting in lipid peroxidation and ferroptosis in AML cells [178]. Similarly, **Mn-Zn ferrite nanoparticles** enhanced adriamycin sensitivity in drug-resistant CML cells through increased ROS and MDA levels, decreased GSH/GSSG ratio, and GPX4 inhibition [179]. These studies collectively demonstrate that ferroptosis-inducing therapies—alone or in combination with existing agents—represent a promising avenue for future leukemia treatment.

### Lymphoma

Compared with leukemia, therapeutic strategies targeting ferroptosis in lymphoma remain largely at the preclinical stage, with limited available data. Nonetheless, several promising studies have revealed that inducing ferroptosis may represent a novel therapeutic approach for lymphoma subtypes.

**Artesunate (ART),** a semisynthetic derivative of the first-line antimalarial drug *artemisinin*, significantly enhances anticancer effects in lymphoma cell lines as a ferroptotic inducer through weakening GPX4/GSH antioxidant defence system and inducing lipid peroxidation [155]. Notably, ABC DLBCL with constitutively active NF-κB appears more susceptible to ART-regulated ferroptosis, demonstrating ART as a potential therapeutic agent for DLBCL [155]. Furthermore, ART was found to induce ferroptosis in DLBCL by impairing STAT3 signaling, with STAT3 silencing further enhancing ART-induced ferroptosis, indicating STAT3‘s regulatory role in ferroptosis in DLBCL cells [180]. Similarly, **kaempferitrin (KPF)**, a natural flavonoid glycoside, induces ferroptosis in DLBCL cells by increasing intracellular Fe^2 +^ and ROS levels, while suppressing GPX4 and SLC7A11 [160]. **APR-246**, currently in clinical trials for acute myeloid leukemia, is also being explored in lymphoma. Studies on TP53-mutated DLBCL cells and xenograft mouse models demonstrated that APR-246 induces p53-dependent ferritinophagy in DLBCL cells harboring a missense mutation in exon 7 [159]. Interestingly, this effect extends to both wild-type and mutant TP53 DLBCL cells, suggesting broad therapeutic potential for APR-246 in this setting [159]. Another promising agent, **ironomycin**, targets lysosomal iron and induces ferroptosis in DLBCL cells by promoting ferritin degradation, reducing cytoplasmic iron, and generating ROS—effects partially blocked by ferrostatin-1 [158].

Several FDA-approved therapies targeting lymphoma have demonstrated a unexpected ferroptotic effects. **Rituximab,** a first-line targeted therapy agent for DLBCL, not only improves clinical outcomes but also induces ferroptosis in OCI-LY1 cells by suppressing the SLC7A11/GPX4 axis, depleting GSH, and increasing MDA levels [156]. Notably,rituximab-resistant cell lines fail to undergo ferroptosis; however, the combining RSL3 with rituximab synergistically overcomes acquired resistance and enhances anticancer efficacy in DLBCL cells, supporting ferroptosis as a therapeutic target for both sensitive and resistant DLBCL cells [156]. Similarly, the combination of the BTK inhibitor **zanubrutinib** with the BCL-2 inhibitor **navitoclax** synergistically suppresses DLBCL progression, particularly in double-hit lymphoma (DHL) [157]. This combination induces both apoptosis and ferroptosis through downregulation of NRF2 and HMOX1 and deactivation of GPX4, supporting ferroptosis as a potential co-target in aggressive DLBCL.

Ferroptosis has also been implicated in natural killer/T-cell lymphoma (NK/TCL). **Kayadiol**, a diterpenoid extracted from *Torreya nucifera*, exerts a significant killing effect on NK/TCL by inducing ferroptosis, as evidenced by ROS accumulation and GSH depletion [181]. These effects are reversed by the ROS scavenger N-acetylcysteine (NAC), GSH, or ferrostatin-1, confirming ferroptotic involvement. Kayadiol also downregulates SLC7A11 and GPX4, with p53 knockout restoring their expression, indicating that the p53/SLC7A11/GPX4 axis plays a key role in kayadiol-induced ferroptosis in NK/TCL cells [181]. Lin et al. [161] further reported that **decitabine plus gemcitabine** treatment decreases SLC7A11 expression in NK/TCL cells, although whether this combination directly triggers ferroptosis remains to be determined.

Nanomedicine-based approaches are also emerging. **HDL-like nanoparticles (HDL NPs)**, which mimic high-density lipoprotein, have shown selective ferroptotic activity in cholesterol uptake–addicted lymphoma cells [182]. HDL NPs trigger compensatory upregulation of cholesterol biosynthesis accompanied by marked GPX4 suppression and lipid peroxidation, culminating in ferroptotic cell death.

Despite these encouraging findings, ferroptosis-targeting strategies in lymphoma are still at an early stage. Future studies should aim to clarify subtype-specific susceptibilities, identify predictive biomarkers, and evaluate combination regimens that can exploit ferroptotic vulnerabilities in resistant or refractory lymphoma.

### Multiple myeloma

Compared with leukemia and lymphoma, research on ferroptosis in MM remains in its early stages, with current findings largely confined to preclinical models. Nonetheless, growing evidence indicates that ferroptosis induction represents a promising therapeutic strategy for MM.Emerging synthetic agents have provided additional insights into ferroptosis regulation in MM.

Emerging synthetic agents have provided additional insights into ferroptosis regulation in MM. **Fingolimod (FTY720)**, a novel immunosuppressant, induces death in MM cells, an effect that can be reversed by ferroptosis-specific inhibitors deferoxamine mesylate (DFOM) and Fer-1. Mechanistically, FTY720 reduces GPX4 and SLC7A11 levels by activating protein phosphatase 2A (PP2A), which dephosphorylates the AMP-activated protein kinase α subunit (AMPKα) at the Thr172 site, subsequently reducing phosphorylated eukaryotic elongation factor 2 (eEF2), finally leading to MM ferrotposis. Moreover, the PP2A inhibitor LB-100 and the AMPK activator AICAR reverses these effects, comfirming the PP2A/AMPK pathway’s role in FTY720-induced ferroptosis [167]. **DCG066,** a novel methyltransferase inhibitor, induces ferroptosis in MM by reducing SLC7A11, GPX4, NRF2, and HO-1 expression, with its effects reversible by ferroptosis inhibitors and the NRF2 activator tert-butyl hydroquinone (TBHQ) [168]. The AP-1 inhibitor **T-5224** suppresses MM growth both in vitro and in vivo by downregulating GPX4 and SLC7A11, effects that are reversed by ferrostatin-1, confirming a ferroptotic mechanism. Mechanistically, *T*-5224 acts through inhibition of the PI3K/AKT pathway, and its combination with bortezomib (BTZ) synergistically enhances cytotoxicity [169].

Several established pharmaceuticals are emerging as potential ferroptosis inducers. **Adapalene(ADA),** a third-generation retinoid used for acne vulgaris and also reported as an avant-garde treatment for several cancers [183–185], accelerates the demise of MM cells by targeting their compensatory survival mechanisms in a dose-dependent manner, and ADA has emerged as a versatile instigator of both ferroptosis and apoptosis in MM cells. Consequently, ADA demonstrates a comprehensive ability to orchestrate MM cell death and represents a novel target for the treatment of MM [186]. Bortezomib (BTZ), a first-line proteasome inhibitor, has recently been shown to trigger ferroptosis by upregulating nuclear receptor coactivator 4 (NCOA4) via inhibition of proteasomal degradation. Combining BTZ with the GPX4 inhibitor RSL3 synergistically enhances ferroptosis in MM cell lines and primary MM samples [120].

Several natural or repurposed compounds have also been shown to induce ferroptosis in MM. **Andrographolide (Andro)**, a diterpenoid lactone derived from *Andrographis paniculata*, is undergoing clinical trials for various cancers [187]. In MM cells, Andro induces oxidative stress, increasing Fe^2 +^ production and lipid peroxidation, which can be rescued by ferroptosis inhibitors. Andro also inhibits the NRF2/HO-1 signaling pathway via activating p38, with p38 inhibition reversing these effects, indicating that Andro induces ferroptosis through the p38/NRF2/HO-1 pathway [162]. **Shikonin (SHK)**, a major bioactive component found in the roots of *Lithospermum erythrorhizon*, exhibits dual functions in MM cells as a proteasome inhibitor and necroptosis inducer, and Li et al. [163] reported that SHK induced ferroptosis in MM by evoking oxidative stress, enhancing ferrous iron levels, and increasing lipid peroxidation, in which treatment with ferroptosis inhibitors reversed SHK-induced cell death. Similarly, **Eclipta prostrata (EEEP)** induces ferroptosis in MM cells by regulating the KEAP1/NRF2/HO-1 axis [164]. **Nitidine chloride (NC)**, extracted from the *Zanthoxylum nitidum*, exhibits ferroptosis-related anti-MM properties both in vitro and in vivo. NC increases intracellular ROS levels, and its effects are reversible with ferroptosis inhibitors. Furthermore, NC directly interacts with ATP-binding cassette sub-family B member 6 (ABCB6), a protein associated with MM relapse, suppressing the PI3K/AKT pathway and promoting ferroptosis, suggesting ABCB6 as a potential therapeutic target and NC as a promising MM treatment candidate [165]. **Apigenin** is one of the most abundant dietary flavonoids and possesses multiple bioactive functions that exhibit anti-myeloma effects through a ferroptotic mechanism, however, apoptosis and autophagy are also observed [188]. **ART** also induces ferroptosis in MM through overexpressing ACSL4 and reducing GPX4 expression, furthermore, ART inhibits the nuclear localization of sterol regulatory element-binding protein 2 (SREBP2), which is accompanied by downregulation of isopentenyl pyrophosphate (IPP) and GPX4, ultimately triggering ferroptosis in myeloma cells [166]. While these compounds remain in preclinical studies, further research is needed for clinical application.

Collectively, these findings underscore the growing recognition of ferroptosis as a critical vulnerability in MM. However, current evidence is limited to preclinical studies. Future work should focus on clarifying the interplay between ferroptosis and conventional MM treatments, identifying predictive biomarkers of ferroptotic sensitivity, and developing clinically applicable ferroptosis-based therapeutic strategies.

## Conclusion and perspectives

In recent years, ferroptosis has emerged as a prominent area of cancer research, particularly in hematologic malignancies, where significant progress has been made. As a newly recognized form of RCD, ferroptosis is uniquely characterized by iron-dependent overwhelming accumulation of lipid peroxides, distinguishing it from apoptosis or autophagy. This process is influenced by a complex network of enzyme, protein, pathway, or organelle participates in the lipid peroxidation. Hematological malignancies, known for their heterogeneity, poor prognosis, and high recurrence rates, pose significant therapeutic challenges. However, recent advances in our understanding of their pathophysiology, coupled with the development of more effective therapies, have transformed these diseases from being largely incurable to manageable conditions.The emerging role of ferroptosis in hematologic cancers has provided new therapeutic perspectives, opening up avenues for novel treatment strategies.

In this review, we summarize the latest studies investigating the mechanisms of ferroptosis in hematological malignancies and potential therapeutic approaches. Despite promising findings demonstrating its therapeutic potential, research on ferroptosis in hematologic malignancies remains in early stages. Ferroptosis regulation varies across different hematologic malignancies, yet its precise mechanisms remain incompletely understood. Targeting specific regulators (e.g., GPX4, SLC7A11, and ACSL4) and exploiting metabolic vulnerabilities (iron metabolism and PUFA-lipid synthesis) provide disease-specific opportunities for precision therapy. However, the lack of reliable biomarkers hampers patient selection and response monitoring, limiting precision medicine applications.

Several ferroptosis inducers—including erastin, RSL3, and various natural compounds—have demonstrated potent anti-tumor activity in preclinical hematologic models. Combination approaches targeting multiple death pathways, such as ferroptosis with apoptosis or autophagy inhibition, have shown synergistic potential and may help overcome drug resistance. Nonetheless, systemic toxicity remains a major concern due to the oxidative nature of ferroptosis, and comprehensive evaluations of pharmacokinetics, safety, and tolerability are still lacking. Moreover, most studies rely on cell lines or xenograft models, with minimal validation in clinical settings.

Future research should prioritize a deeper mechanistic understanding of ferroptosis across diverse hematologic malignancies, including systematic mapping of context-specific regulators and pathways. Particular attention should be given to underexplored molecules such as GCH1 and FSP1, which may serve as key modulators or therapeutic targets. Identifying predictive biomarkers will be essential for stratifying patients and monitoring therapeutic responses. Parallel efforts should aim to design safer ferroptosis-based interventions through improved drug formulations, targeted delivery systems, and rational combination strategies that mitigate adaptive resistance via antioxidant pathway upregulation or compensatory survival signaling.

In conclusion, ferroptosis represents a transformative therapeutic opportunity in hematologic malignancies. By addressing current limitations and prioritizing mechanistic studies, biomarker identification, and safe clinical translation, ferroptosis-targeting therapies could become an effective strategy to overcome drug resistance and improve outcomes in patients with aggressive or refractory hematologic cancers.

## Data Availability

No datasets were generated or analysed during the current study.
